# Self-management using crude herbs and the health-related quality of life among adult patients with hypertension living in a suburban setting of Malaysia

**DOI:** 10.1371/journal.pone.0257336

**Published:** 2021-09-10

**Authors:** Raphael Joe Joachimdass, Kavitha Subramaniam, Nam Weng Sit, Yang Mooi Lim, Chin Hai Teo, Chirk Jenn Ng, Afzaninawati Suria Yusof, Annaletchumy Loganathan

**Affiliations:** 1 Faculty of Science, Universiti Tunku Abdul Rahman, Kampar, Malaysia; 2 Department of Pre-Clincal Science, Universiti Tunku Abdul Rahman, Sungai Long, Malaysia; 3 Centre for Cancer Research, Faculty of Medicine and Health Sciences, Universiti Tunku Abdul Rahman, Kajang, Selangor, Malaysia; 4 Faculty of Medicine, University of Malaya, Kuala Lumpur, Malaysia; 5 Perak State Health Department, Kementerian Kesihatan Malaysia, Ipoh, Malaysia; Endeavour College of Natural Health, AUSTRALIA

## Abstract

**Purpose:**

To determine the prevalence of crude herbs’ use in the self-management of hypertension and the health-related quality of life (HRQOL) in patients with hypertension.

**Methods:**

This cross-sectional study was performed among patients with hypertension attending a government health clinic. Socio-demographic characteristics, lifestyle modifications, medical history and predictors of crude herbs users were obtained. The diversity of crude herbs used was assessed using a modified international complementary and alternative medicine questionnaire (I-CAM-Q) and the HRQOL was assessed using the SF36 instrument.

**Results:**

Out of the 294 patients recruited, 52.4% were female, 41.5% were Malay and 38.8% were within the 60 to69 age category. The prevalence of crude herbs users was 30.6% and the most common herbs used were pegaga (*Centella asiatica)*, peria (*Momordica charantia)* and betik (*Carica papaya)*. Using the regression analysis, significantly higher odds of using crude herbs are noted among Malay or Indian patients who have these characteristics: attained secondary education, experienced falls or muscle pain, and had systolic blood pressure of more than 140 mmHg. There was no significant difference in HRQOL domains between the crude herb users and non-users (*p*>0.05).

**Conclusion:**

Besides taking allopathic medications, certain patients with hypertension use crude herbs as a form of self-management. Although patients are adamant about integrating crude herbs as a form of self-management, the effects of doing so have not been properly investigated. This implies that the healthcare staff members need to communicate with the patients regarding the use of crude herbs together with conventional drugs.

## Introduction

Hypertension is a major public health issue, with 1.13 billion people diagnosed with the disease worldwide in 2019 [1]. This condition if left untreated increases the risk of several other chronic ailments such as cardiovascular diseases, kidney failure and stroke [2]. Based on the National Health and Morbidity Survey (NHMS) conducted in Malaysia in 2019, the overall prevalence of hypertension was 30.0% showing a slight reduction from the 30.3% obtained in 2015 [3]. With this high figure, the proper management of hypertension among Malaysian patients remains a difficult task. A previous study indicated that most diagnosed patients undergo treatment for hypertension, but only one quarter of them have their blood pressure under control [4]. This could be attributed to several barriers such as non-adherence to medication, lack of counselling from physicians, and the worry of being on life-long medication [5].

Numerous methods encompassing conventional therapies and non-conventional interventions have been developed with the purpose of managing hypertension. In the conventional therapy, drug classes such as angiotensin-converting enzyme (ACE) inhibitors, beta-blockers, thiazides, calcium channel blockers and alpha-blockers are commonly prescribed to reduce blood pressure levels in hypertensive patients [6]. The non-conventional interventions which have been proven effective in managing hypertension include dietary changes such as reduction of sodium chloride intake and increased physical activities resulting in weight loss [7]. Moreover, a systematic review of lifestyle interventions stresses that limited alcohol intakes and controlled diet strategies are effective for decreasing blood pressure [8].

In recent years, patients have begun to use complementary and alternative medicine (CAM) to self-manage chronic diseases [8]. With regard to managing hypertension, patients have used slow breathing techniques, Qigong, consumption of dark chocolate, and meditation [9]. Another type of CAM increasingly used for hypertension management involves crude herbs (i.e., raw plants that have not undergone drying or other types of processing). The prevalence of crude herbs use as indicated in previous studies ranges from 12.8% to 69% [10–18]. A study in Ghana indicated that patients used moringa (*Moringa oleifera*), bitter leaf (*Vernonia amygdalina*), garlic (*Allium sativum*), dandelion (*Taraxacum officinale*), cotton plant (*Gossypium*), or a combination of two or more plants to manage their hypertension [17]. In a Malaysian study, herbal products used by patients with hypertension include misai kucing (*Orthosiphon stamineus*), bitter gourd (*Momordica charantia*), basil (*Ocimum basilicum*), ginseng (*Panax ginseng*), and garlic (*Allium sativum*) [19]. Meanwhile, a Jamaican study found that patients with hypertension took moringa, ginger, lime, garlic (*Allium sativum*), and guinea hen weed (*Petiveria alliacea*) [20]. Patients took crude herbs for several reasons, including relieving symptoms of hypertension and lowering blood pressure, as well as the perception that conventional medicine did not work [13, 14]. Other studies reported significant associations between use of crude herbs and factors such as education level, age, and having other family members with hypertension [11, 13, 14].

The concept of health-related quality of life (HRQOL) has gained interest in health and clinical research in recent years [21, 22]. Furthermore, HRQOL has emerged as an essential outcome in studies related to hypertension, as it may serve as an indicator to evaluate antihypertensive treatment outcomes [22, 23]. Previous findings indicate that hypertensive patients who consumed herbs or herbal-based products were reported to have low or middle levels of quality of health [22]. In contrast, a group of researchers concluded that a short yoga programme carried out by hypertensive patients showed blood pressure lowering effects and positive outcomes of self-rated quality of life [24].

The use of crude herbs to treat hypertension is notable in numerous developed and developing countries. Malaysia has also been recorded as one of the top bio-diverse countries with about 23,000 documented plant species, the use of which among patients with chronic diseases remains unexplored [25]. There is a lack of studies that emphasise the patients’ use of crude herbs for managing chronic diseases, although the use of herbs is extremely widespread and engraved in the cultural norms of the Malaysian people. Therefore, this study aims to fill the gap by investigating the self-management of hypertension using herbs that includes identifying the predictors, the diversity of crude herbs used, and comparing the HRQOL between crude herbs users and non-users that reside in a suburban setting of Malaysia.

## Methods

### Study design, site and patients

A cross-sectional study design is employed for this study. The study was conducted in Klinik Kesihatan Kampar, a primary healthcare clinic situated in the suburban district of Kampar. This government healthcare facility provides primary healthcare for residents residing in nearby areas. The study was conducted in six months from October 2019 to March 2020. Adults aged 18 and above, diagnosed with hypertension and attended the clinic for at least three prior appointments for hypertension, were included in this study. Patients who were unable to understand and communicate in either Bahasa Melayu or English or mentally or physically impaired were not recruited for this study. The sample size was calculated based on the prevalence rate of crude herbs use of 15.3% as reported by the patients who attended a government health clinic in Ipoh [11, 26], which is located near to the study area. A 95% confidence level and margin of error of 4.5% were used; the sample size determined was 246, which was rounded to 300 patients.

### Data collection tool

The questionnaire consists of socio-demographic characteristics, lifestyle modifications, medical history, HRQOL indices; besides, blood pressure measurements of the participants were taken. The SF-36 is a questionnaire that consists of 36 questions and explores 9 different health aspects: general health perceptions, role limitations due to emotional or personal problems, role limitations due to physical health issues, bodily pain, physical functioning, energy or fatigue, social functioning, emotional well-being and general health [26]. The crude herbs details were obtained using an adapted version of the International Complementary and Alternative Medicine Questionnaire (I-CAM-Q) [27]. The questionnaire was adapted to obtain information on herbs used only for the hypertensive condition and not for other purposes. The questions are designed to obtain these details about the herbs used: types, parts, preparation methods, frequency of use, current usage, and helpfulness in managing blood pressure. In addition, other related pieces of information were also obtained such as patients’ disclosures of the crude herbs used to their doctor, reasons for use, and sources of recommendation. Findings on the CAM use would be further discussed in a separate publication. The English questionnaire (S1 File) was translated into Bahasa Melayu (BM) (S2 File) by a local BM teacher, and it was tested on a pilot scale among 10 individuals.

### Data collection method

Participants for this study were recruited using the convenience sampling technique. The researchers would be present to collect data at the study site on selected weekdays from Monday to Thursday. Details of the study participants required for this study together with the inclusion and exclusion criteria were explained to the doctors and nurses attending to the patients in Klinik Kesihatan Kampar. If a patient met the inclusion criteria, the doctor would recommend the patient to participate in our survey. Nurses would guide the patients to the study site situated nearby the doctor’s consultation room. The researchers would then brief the patients about the study. Patients who agreed to participate in the survey would complete a consent form, and have their weight, height and blood pressure measurements taken. The participating patients’ blood pressure was measured using an OMRON Automatic Blood Pressure Monitor (HEM-7120). The patients were assured that all collected data would be used for academic research purposes and would remain confidential. The researcher was trained on questionnaire administration by the principal investigator. Patients who understood English or Bahasa Melayu but not fluent in it were aided by a family member or friend during the interview. Missing data from the questionnaire would be obtained subsequently by contacting the patients through messaging apps and telephone calls.

### Ethical considerations

An ethical approval for this study was obtained from Universiti Tunku Abdul Rahman Scientific and Ethical Review Committee (U/SERC/207/2019), National Medical Research Register (NMRR-17-2591-38273), and Klinik Kesihatan Kampar. Patients’ written consent was obtained using the Volunteer Information and Consent Form (FM-IPSR-R&D-057) and Personal Data Protection Statement from Universiti Tunku Abdul Rahman.

### Data analysis

The SPSS version 25 was used to analyse the data collected from the cross-sectional study. Data obtained from the face-to-face interview were first entered into the SPSS version 25. The descriptive statistics were presented as mean ± standard deviation, frequencies, or percentages. The Chi-square test was performed to find out the association between the socio-demographic characteristics, basic measurements, medical history and lifestyle modifications, with the patients’ use of crude herbs. Fishers exact test was used to study the association between race and the use of crude herbs due to small sample size for the ‘others’ race group. The predictors of crude herbs use by the hypertensive patients were determined using the multiple logistic regression analysis. The Mann-Whitney U test was applied to compare the distribution for HRQOL domains among crude herbs users and non-users. A p-value <0.05 was used as the critical value for statistical significance.

## Results

### Socio-demographic characteristics and basic measurements

Out of the 306 patients approached, 294 participated in this study, representing a 96% response rate. Some patients cited lack of time as the main reason for not participating in the study. The 60 to 69 age category was the most frequent (n = 114, 38.8%). Most of the patients were Malay (n = 122, 41.5%), followed by Chinese (n = 115, 39.1%). A large percentage of the patients did not have monthly income (n = 178, 60.5%), and most patients attained secondary education and higher (n = 151, 51.4%). A total of 114 (38.8%) patients were housewives, while 101 (34.4%) were retired. More than half of the patients were diagnosed with hypertension for at least 5 years (n = 165, 56.1%). A total of 117 (39.8%) patients had uncontrolled hypertension (systolic blood pressure ≥ 140 mmHg and/or diastolic blood pressure ≥ 90 mmHg). For body mass index, most of them were normal or underweight (n = 125, 42.5%) (Table 1).

**Table 1:** The determinants of crude herbs use.

Variables	Total (n = 294)		Taking crude herbs				Chi-square (χ^2^); *p*-value
			Yes (n = 90)		No (n = 204)		
	n	%	n	%	n	%	
**Age groups**							
<50	25	8.5	11	44.0	14	56.0	8.182; 0.042*
50–59	76	25.9	27	35.5	49	64.5	
60–69	114	38.8	37	32.5	77	67.5	
>69	79	26.9	15	19.0	64	81.0	
**Gender**							
Male	140	47.6	34	24.3	106	75.7	5.036; 0.025*
Female	154	52.4	56	36.4	98	63.6	
**Race**							
Malay	122	41.5	59	48.4	63	51.6	49.963; 0.000*^a^
Chinese	115	39.1	10	8.7	105	91.3	
Indian	48	16.3	17	35.4	31	64.6	
Others	9	3.1	4	44.4	5	55.6	
**Monthly Income**							
No income	178	60.5	43	24.2	135	75.8	8.876; 0.012*
<RM 3000	98	33.3	40	40.8	58	59.2	
>RM 3000	18	6.1	7	38.9	11	61.1	
**Education level**							
No formal education	37	12.6	11	29.7	26	70.3	11.985;
Primary education	106	36.1	20	18.9	86	81.1	0.002*
Secondary education or higher	151	51.4	59	39.1	92	60.9	
**Employment status**							
Working Full time	58	19.7	19	32.8	39	67.2	3.588; 0.309
Working Part time	19	6.5	6	31.6	13	68.4	
Housewife	116	39.5	40	35.1	74	64.9	
Unemployed	2	0.7	1	50	1	50	
Retired	101	34.4	24	23.8	77	76.2	
**Duration of Hypertension**							
<5 years	129	43.9	39	30.2	90	69.8	0.016; 0.901
>5 years	165	56.1	51	30.9	114	69.1	
**Systolic Blood Pressure**							
<140 mmHg	185	62.93	47	25.4	138	74.6	6.369; 0.012*
≥140 mmHg	109	37.07	43	39.4	66	60.6	
**Diastolic Blood Pressure**							
<80 mmHg	147	50.0	39	26.5	108	73.5	2.320; 0.314
80–89 mmHg	100	34.0	35	35.0	65	65.0	
≥90 mmHg	47	16.0	16	34.0	31	66.0	
**Body Mass Index**							
< 24.9 kg/m^2^	125	42.5	37	29.6	88	70.4	3.835; 0.147
25–29.9 kg/m^2^	101	34.4	26	25.7	75	74.3	
≥30 kg/m^2^	68	23.1	27	39.7	41	60.3	
**Lifestyle Modifications**							
Trying to reduce body weight	26	8.8	10	38.5	16	61.5	0.827, 0.363
Maintaining healthy body weight	12	4.1	5	41.7	7	58.3	-
Reduce sodium (salt) intake	168	57.1	55	32.7	113	67.3	0.834. 0.361
Reduce/stop alcohol consumption	7	2.4	3	42.9	4	57.1	-
Regular physical activity (at least 90 minutes a week)	115	39.1	41	35.7	74	64.3	2.259,
							0.133
Healthy eating (as advised by doctor)	123	41.8	38	30.9	85	69.1	0.008, 0.929
Reduce/stop smoking	15	5.1	3	20.0	12	80.0	-
Stress management	4	1.4	3	75.0	1	15.0	-
Increased dietary potassium intake	10	3.4	3	30.0	7	70.0	-
**Medical History**							
Asthma	19	6.5	4	21.1	15	78.9	0.874, 0.350
Cancer	5	1.7	3	60.0	2	40.0	-
Cardiovascular disease	28	9.5	12	42.9	16	57.1	2.185
							0.139
Diabetes Mellitus Type-II	90	30.6	34	37.8	56	62.2	3.135, 0.077
Dyslipidemia	182	61.9	62	34.1	120	65.9	2.683, 0.101
Hyperuricemia	5	1.7	4	80.0	1	20.0	-
Kidney disease	2	0.7	1	50.0	1	50.0	-
Liver disease	4	1.4	1	25.0	3	75.0	-
Migraine	53	18.0	18	34.0	35	66.0	0.342, 0.559
Muscle pain	45	15.3	20	44.4	25	55.6	4.786,
							0.029*
Obesity	68	23.1	27	30.0	41	20.1	3.444, 0.063
Parkinson	1	0.3	0	0.0	1	100	-
Peptic ulcer	2	0.7	1	50.0	1	50.0	-
Stroke	4	1.4	3	75.0	1	25.0	-
Thyroid disease	2	0.7	1	50.0	1	50.0	-
Urinary tract infection	4	1.4	2	50.0	2	50.0	-
Falls	23	7.8	14	60.9	9	39.1	10.754, 0.001*
Osteoporosis	1	0.3	0	0.0	1	100	-
**Number of comorbidities**												
None	47	16.0	6	12.8	41	87.2	13.386, 0.004*
1	84	28.6	21	25.0	63	75.0	
2	83	28.2	31	37.3	52	62.7	
3 or more	80	27.2	32	40.0	48	60.0	

*significant association, ‘-‘ indicates that Chi-square analysis was not performed due to insufficient data

a–Fishers exact test was used.

### Lifestyle modifications of patients

The lifestyle modifications opted by the patients with hypertension attending Klinik Kesihatan Kampar are depicted in Table 1. The three most common lifestyle modifications practised were reducing sodium intake (n = 168, 57.1%), healthy eating (n = 123, 41.8%) and regular physical activity (n = 115, 39.1%).

### Medical history of patients

Table 1 details the medical history of patients with hypertension who participated in this study. The five most common comorbidities were dyslipidemia (n = 182, 61.9%), diabetes mellitus type-2 (n = 90, 30.6%), obesity (n = 68, 23.1%), migraine (n = 53, 18.0%) and muscle pain (n = 45, 15.3%). Most of the patients (n = 84, 28.6%) had one comorbidity.

### Diversity of crude herbs used

The prevalence of crude herbs use is 30.6% (n = 90). The number of users for each herb, parts, preparation methods, current use, frequency of use, and helpfulness in managing blood pressure among the patients attending Klinik Kesihatan Kampar is shown in Table 2. There are a total of 52 different combinations of known crude herbs that were reported by the patients. Twenty-one of those crude herbs were taken by three or more users. The five most commonly used herbs consumed by patients are pegaga, *Centella asiatica* (n = 28. 31.11%), peria, *Momordica charantia* (n = 23, 25.56), betik, *Carica papaya* (n = 17, 18.89%), timun, *Cucumis sativus* (n = 16, 17.78%) and petai, *Parkia speciosa* (n = 10, 11.11%).

**Table 2:** Diversity of the herbs used.

Scientific Name (Local Name of Herbs)	Number of Users (n), percentage (%)	Part of Herbs (n)	Preparation Methods (n)	Current Users of Herbs	Frequency of Use (n)	Helpful in managing blood pressure (n)
*Centella asiatica* (pegaga)	28, 31.11%	Leaves or shoots (26), fruits (1), blended into juice (1)	Raw (24), blended into juice (3), infusion (1)	25	Often (17), sometimes (8), rarely (3)	Helpful (17), not helpful (11)
*Momordica charantia* (peria)	23, 25.56%	Fruit (23)	Raw (22), Blended into juice (1)	22	Often (19), sometimes (3), rarely (1)	Helpful (12), not helpful (11)
*Carica papaya* (betik)	17, 18.89%	Leaves (17)	Raw (13), steamed (3), infusion (1)	16	Often (9), sometimes (7), rarely (1)	Helpful (12), not helpful (5)
*Cucumis sativus* (timun)	16, 17.78%	Fruit (16)	Raw (15), Blended into juice (1)	16	Often (13), Sometimes (3)	Helpful (5), Not helpful (11)
*Parkia speciosa* (petai)	10, 11.11%	Seeds (10)	Raw (1)	10	Often (3), Sometimes (7)	Helpful (3), not helpful (7)
*Moringa oleifera* (murungai)	9, 10.00%	Leaves (8) Fruit (1)	Infusion (6) Boiled (1) Raw (1) Blended into juice (1)	9	Often (7) Sometimes (2)	Helpful (9)
*Cosmos caudatus* (ulam raja)	8, 8.89%	Leaves (8)	Raw (8)	8	Often (7), Sometimes (1)	Helpful (2), not helpful (6)
*Momordica charantia* and *Malus domestica* (peria & epal hijau)	7, 7.78%	Fruit (7)	Blended into juice (7)	7	Often (5), Sometimes (2)	Helpful (4), Not helpful (3)
*Oenanthe javanica* (selom)	4, 4.44%	Leaves (4)	Raw (4)	4	Often (3), Sometimes (1)	Helpful (3), not helpful (1)
*Psophocarpus tetragonolobus* (kacang botol)	4, 4.44%	Beans (4)	Raw (4)	4	Often (3) Sometimes (1)	Helpful (2) Not helpful (2)
*Melicope ptelefolia* (tenggek burung)	4, 4.44%	Leaves or shoots (4)	Raw (4)	4	Often (4)	Helpful (3), Not helpful (1)
*Anacardium occidentale* (gajus)	4, 4.44%	Leaves or shoots (4)	Raw (3) Steamed (1)	4	Often (4)	Helpful (4)
*Malus domestica* (epal hijau)	4, 4.44%	Fruit (4)	Blended into juice (3), raw (1)	4	Often (3), Sometimes (1)	Helpful (2), Not helpful (2)
*Daucus carota subsp*. *Sativus* (lobak merah)	4, 4.44%	Fruit (4)	Blended into Juice (2), raw (2)	4	Often (1) Sometimes (3)	Helpful (1), Not helpful (3)
*Apium graveolens* (saderi)	3, 3.33%	Leaves or shoots (3)	Raw (1) Infusion (1) Boiled (1)	3	Often (3)	Helpful (3)
*Clinacanthus nutans* (belalai gajah)	3, 3.33%	Leaves (3)	Raw (3)	3	Sometimes (2), rarely (1)	Helpful (2), not helpful (1)
*Manihot esculenta* (ubi kayu)	3, 3.33%	Leaves (3)	Raw (2) Steamed (1)	3	Often (3)	Helpful (1), not helpful (2)
*Brassica oleracea* (kubis)	3, 3.33%	Leaves (3)	Raw (3)	1	Often (2), Sometimes (1)	Helpful (1), Not helpful (2)
*Mentha arvensis* (pudina)	3, 3.33%	Leaves (3)	Infusion (3)	3	Often (1), Sometimes (2)	Helpful (1), Not sure (2)
*Cuminum cyminum* (jintan putih)	3, 3.33%	Seeds (3)	Infusion (3)	3	Often (2), Sometimes (1)	Helpful (3)
*Lactuca sativa* (salad)	3, 3.33%	Leaves (3)	Raw (3)	3	Often (3)	Helpful (3)
*Citrus aurantiifolia* (limau nipis)	2, 2.22%	Fruit (2)	Blended into juice (2)	2	Sometimes (2)	Helpful (1), Not helpful (1)
*Phaleria macrocarpa* (mahkota dewa)	2, 2.22%	Fruit (1) Leaves (1)	Raw (1) Infusion (1)	2	Sometimes (2)	Helpful (2)
*Mangifera indica (manga)*	2, 2.22%	Leaves (1) Fruits (1)	Raw (2)	2	Sometimes (2)	Helpful (2)
*Eurycoma longifolia* (tongkat ali)	2, 2.22%	Roots (2)	Boiled (1), infusion (1)	2	Sometimes (1), rarely (1)	Helpful (2)
*Psidium guajava* (jambu batu)	2, 2.22%	Leaves (2)	Boiled (1), infusion (1)	2	Sometimes (2)	Helpful (2)
*Orthosiphon aristatus* (misai kucing)	2, 2.22%	Leaves (1), Leaves and root (1)	Boiled (2)	2	Sometimes (1), rarely (1)	Helpful (2)
*Tamarindus indica* (asam jawa)	2, 2.22%	Fruit (1), Leaves (1)	Infusion (2),	2	Often (2)	Helpful (2)
*Piper sarmentosum* (daun kaduk)	1, 1.11%	Leaves (1)	Raw (1)	1	Sometimes (1)	Helpful (1)
*Morinda citrifolia* (mengkudu)	1, 1.11%	Fruit (1)	Raw (1)	1	Sometimes (1)	Helpful (1)
*Brassica chinensis* (sawi putih)	1, 1.11%	Leaves (1)	Raw (1)	1	Often (1)	Helpful (1)
*Musa paradisiaca* (pisang)	1, 1.11%	Flower (1)	Steamed (1)	1	Often (1)	Helpful (1)
*Prunus avium* (ceri)	1, 1.11%	Leaves (1)	Steamed (1)	1	Sometimes (1)	Helpful (1)
*Andrographis paniculata* (hempedu bumi)	1, 1.11%	Leaves (1)	Steamed (1)	1	Sometimes (1)	Helpful (1)
*Mitragyna speciosa* (ketum)	1, 1.11%	Leaves (1)	Raw (1)	0	Rarely (1)	Not Helpful (1)
*Phyllanthus acidus* (gooseberry)	1, 1.11%	Fruit (1)	Raw (1)	1	Sometimes (1)	Helpful (1)
*Diplazium esculentum* (sayur paku)	1, 1.11%	Leaves (1)	Raw (1)	1	Sometimes (1)	Helpful (1)
*Allium sativum* (bawang putih)	1, 1.11%	Bulb (1)	Raw (1)	1	Often (1)	Not Helpful (1)
*Murraya koenigii* (daun kari)	1, 1.11%	Leaves (1)	Raw (1)	1	Often (1),	Not helpful (1)
*Chrysanthemum morifolium* (bunga kekwa)	1, 1.11%	Flowers (1)	Infusion (1)	1	Rarely (1)	Not helpful (1)
*Solanum torvum* (terung pipit)	1, 1.11%	Fruit (1)	Raw (1)	1	Often (1)	Not helpful (1)
*Coriandrum sativum* (daun ketumbar)	1, 1.11%	Leaves (1)	Infusion (1)	1	Often (1)	Helpful (1)
*Solanum lycopersicum* (tomato)	1, 1.11%	Fruit (1)	Raw (1)	1	Often (1)	Helpful (1)
*Vigna unguiculata* (asparagus)	1, 1.11%	Fruit (1)	Raw (1)	1	Sometimes (1)	Not helpful (1)
*Citrus limon* (limau)	1, 1.11%	Fruit (1)	Infusion (1)	1	Often (1)	Not helpful (1)
*Actinidia deliciosa* (kiwi)	1, 1.11%	Fruit (1)	Raw (1)	1	Often (1)	Not helpful (1)
*Vitis vinifera* (anggur)	1, 1.11%	Fruit (1)	Raw (1)	1	Often (1)	Not helpful (1)
*Citrus reticulata* (mandarin)	1, 1.11%	Fruit (1)	Raw (1)	1	Rarely (1)	Helpful (1)
*Trigonella foenum-graecum* (fenugreek)	1, 1.11%	Leaves (1)	Infusion (1)	1	Often (1)	Helpful (1)
*Abelmoschus esculentus* (bendi)	1, 1.11%	Fruit (1)	Infusion (1)	1	Often (1)	Helpful (1)
*Malus domestica and Cucumis sativus* (epal hijau & timun)	1, 1.11%	Fruit (1)	Blended into juice (1)	1	Often (1)	Not helpful (1)
*Vernonia amygdalina* (bitter leaf)	1, 1.11%	Leaves (1)	Infusion (1)	1	Sometimes (1)	Helpful (1)

*Frequency of use: Often (≥ three times a month), Sometimes (once or twice a month), Rarely (less than twice in three months), n: number of users. Helpfulness in managing blood pressure is based on the user’s perception of how the crude herbs helped them in hypertension control.

### Predictors of crude herbs use

The association between the use of herbs and sociodemographic characteristics, BMI or blood pressure measurements was determined using chi-square analysis as shown in Table 1. Crude herbs use was significantly associated with gender (female) (χ2 = 5.036, *p* = 0.025); race (Malay and Indian and others) (χ2 = 49.963, *p* = 0.000); monthly income (no income) (χ2 = 8.876, *p* = 0.012); and education level (secondary education) (χ2 = 11.985, *p* = 0.002). Furthermore, the systolic blood pressure (χ2 = 6.369, *p* = 0.039) was significantly associated with the use of crude herbs. For medical history, muscle pain (χ^2^ = 4.786, *p* = 0.029), falls (χ^2^ = 10.754, *p* = 0.001), and the number of comorbidities (χ^2^ = 13.386, *p* = 0.004) showed significant association.

Using the multivariate logistic regression analysis, several predictors of crude herbs use were determined as shown in Table 3. Patients who were Malay (OR: 8.646, 95% C.I.: 4.011–18.637) and patients who were Indian (OR: 4.433, 95% C.I.: 1.757–11.183) had higher odds of taking crude herbs when compared with the Chinese patients. Experiencing falls (OR: 3.011, 95% C.I.: 1.110–8.169) and having muscle pains (OR: 2.227, 95% C.I.: 1.035–4.792) were also predictors of crude herbs use. For systolic blood pressure, patients who had a reading of 140 mmHg or more (OR: 2.389, 95% C.I.: 1.311–4.353) had higher odds of consuming crude herbs when compared with patients with systolic reading of less than 140 mmHg. In terms of education level, patients with secondary education or higher (OR: 2.783, 95% C.I.: 1.433–5.402), had higher odds of taking crude herbs as compared with patients having only primary education.

**Table 3:** Predictors of crude herbs use.

Variable	*p*-value	OR	95% C.I.
			Lower	Upper
**Falls**				
Yes	0.030	3.011	1.110	8.169
No*				
**Muscle Pain**				
Yes	0.041	2.227	1.035	4.792
No*				
**Ethnicity**				
Malay	0.000	8.646	4.011	18.637
Chinese*				
Indian	0.002	4.433	1.757	11.183
Others	0.069	4.651	0.0886	24.415
**Education level**				
No formal education	0.193	1.906	0.722	5.030
Primary education*				
Secondary education or higher	0.002	2.783	1.433	5.402
**Systolic Blood Pressure**				
<140 mmHg*				
≥140 mmHg	0.004	2.389	1.311	4.353

*reference category.

### HRQOL measurements

**Fig 1:**
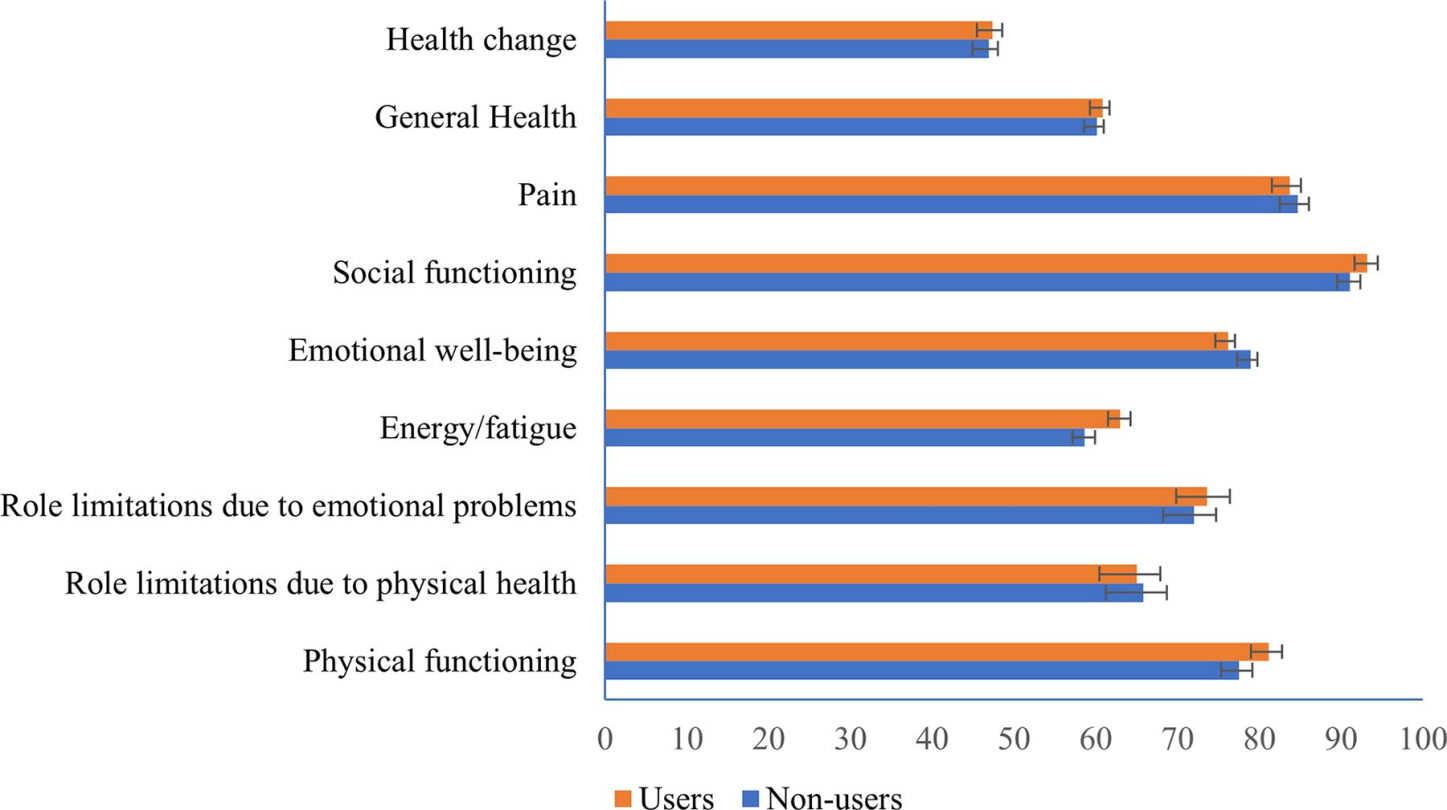
Mean scores of HRQOL domains for crude herb users and non-users.

Based on S1 Table, the HRQOL domain with the highest mean score is social functioning (91.72 ± 17.13) while the lowest score is health change (41.07 ± 17.05). The mean scores of physical functioning, role limitations due to emotional problems, energy or fatigue, social functioning, general health and health change domains were higher in patients who took crude herbs than those who did not take crude herbs. On the other hand, the mean scores for role limitations due to physical health, emotional well-being and pain were higher in non-users than users. Social functioning has the highest mean score of among all HRQOL domains for both crude herbs users (93.24 ± 14.84) and non-users (91.05 ± 18.05). Similarly, the HRQOL domain with the lowest mean score was health change for both crude herbs users (47.40 ± 18.60) and non-users (46.89 ± 16.37) (Fig 1). The Mann-Whitney’s U test showed that there was no significant difference (p>0.05) between crude herbs users and non-users for all HRQOL domains.

### Disclosure of crude herbs use with doctor

The majority of crude herbs users (n = 84, 93.33%) did not inform their doctor of the use of crude herbs. The reasons why the patients chose not to tell their doctors about their use of crude herbs are shown in Table 4. There are three main reasons given by the patients: the doctor did not ask (79.76%, n = 67); felt that eating herbs was normal (7.14%, n = 6); and no particular reason (7.14%, n = 6).

**Table 4:** Patient’s reason for not informing doctor about herbs usage, reason for taking crude herbs and sources of recommendation.

	Frequency	Percentage (%)
**Reason for not informing doctor about herbs use**		
The doctor did not ask	67	79.76
No particular reason	6	7.14
Felt that eating herbs was normal	6	7.14
Unsure about the herb’s effects	5	5.95
Feared that the doctor might scold them	2	2.38
Did not feel the need to tell the doctor	2	2.38
**Reason for taking crude herbs**		
Easily accessible	52	57.78
Cultural reasons	51	56.67
Traditional beliefs	31	34.44
Appetising	9	10.00
Listen to friends or family’s advice	6	6.67
Help to further reduce blood pressure	4	4.44
To maintain good health	2	2.22
Religious beliefs	2	2.22
Worried about their health	2	2.22
To cleanse the body	1	1.11
Dissatisfied with allopathic medication	1	1.11
As prevention for diseases	1	1.11
**Source of Recommendation**		
Family	75	83.3
Friends	44	48.9
Internet	11	12.2
Newspaper	10	11.1
Television	7	7.8
Book	5	5.6
Store clerk	1	1.1
Pharmacist	1	1.1

### Reasons for taking crude herbs

Among the crude herbs users, there are several factors that cause them to consume crude herbs, as depicted in Table 4. The five main reasons are as follows. First, most crude herbs users felt that the herbs were easily accessible (57.78%, n = 52). Second and third, some users cited cultural reasons (56.67%, n = 51), and traditional beliefs (34.44%, n = 31) for their use of crude herbs. Fourth, a few users said that herbs were appetising (10%, n = 9); and fifth, some claimed that they took herbs after listening to friends’ or family’s advice (6.67%, n = 6).

### Sources of recommendation

The most common source of recommendation for herbs usage is family (83.3%, n = 75), as indicated in Table 4. This is followed by friends (48.9%, n = 44) and the Internet (12.2%, n = 11). These figures indicate that crude herbs users are mainly persuaded by family and friends to try crude herbs.

## Discussion

This cross-sectional survey determined the prevalence and predictors of crude herbs integration among the patients with hypertension that attended a government health clinic located in a suburban setting. The prevalence of crude herbs use is 30.6%. This suggests that despite receiving primary care for hypertension, the patients recruited in this study resorted to crude herbs for self-managing blood pressure. On the other hand, the predictors of crude herbs use are as follows: being Malay or Indian, having secondary education, having muscle pain, experiencing falls, and having a systolic blood pressure of 140 mmHg or higher. Crude herbs users showed no significant associations for all HRQOL domains.

In this study, the prevalence of crude herbs use is 30.6%—double the prevalence of herbal medicine usage in a previous study conducted in an urban primary health clinic in Malaysia [11]. Other previous studies conducted in the Asian countries reported varying prevalence: Palestine (62.1%) [14], China (18.5%) [28], and Iran (29.4%) [29]. The different prevalence noted could be attributed to these factors: availability and diversity of herbs species across countries with different climate; varied cultural practices of people with different ethnicities; and different definitions of herbs used in the previous studies.

This study found that the Malay and Indian are predictors of crude herbs use. Cultural and religious factors may play a part because more Malays and Indians consume crude herbs as compared with the Chinese population. The Malays consume “ulam” or salad as a staple in their culture [30]. Ulam consists of traditional plants found in Malaysia and are usually taken as part of a meal, either raw or blanched [30]. From the perspective of religion, a previous study, done by Ching and colleagues in a government health clinic in Sepang, Malaysia, identified that being Muslim was a predictor of herbs use for type 2 diabetes mellitus [19]. In Malaysia, virtually all Malays embrace the Islam religion. A study performed in the Middle East attributed the use of medicinal herbs to Islamic beliefs and culture; the Muslims in the region believe in the use of herbs as treatment for numerous health conditions [31]. In Malaysia, the Indian community is accustomed to using herbs for many purposes such as well-being, treating diseases, cooking, and celebrations during festivals; these practices can be traced back to the ancient times [32].

Patients with secondary education and higher are shown to be a predictor of crude herbs use. As Malaysia is considered a developing country, this finding is in line with those of other CAM studies performed in more developed countries like Turkey [33] and China [34]. On the other hand, a Palestinian study found that those with lower education level are significantly associated with crude herbs use [14]. Having muscle pains and experiencing falls in the past 6 months are predictors of crude herbs use. In a previous qualitative investigation involving elderly persons who had experienced falls, several subjects took traditional medicine as a form of self-care and they found this form of treatment effective [35]. A review indicates that herbs such as saffron and garlic have been applied in the treatment and prevention of muscle soreness [36]. People have also sought out herbal therapies to alleviate inflammation, muscle soreness and pains [36]. The fact that patients who had falls and muscle pains were identified as predictors in this study may indicate that patients could have taken herbs to cope with multiple ailments in addition to their hypertensive condition. Another key finding in this study is that patients with higher systolic blood pressure had significantly higher odds of taking crude herbs compared with patients with lower systolic blood pressure. This could mean patients who seem unable to reduce their systolic blood pressure to a normal range would choose to integrate herbs as a complementary therapy.

From this study, some of the more prominently used crude herbs by patients are *Centella asiatica*, *Momordica charantia*, *Carica papaya*, *Cucumis sativus*, *Parkia speciosa*, *Moringa oleifera* and *Cosmos caudatus*. In two previous surveys performed locally, *Momordica charantia*, *Centella asiatica* and *Carica papaya* are similar to some of the main herbs used by patients with hypertension [11, 29]. This common finding could be due to the availability of and accessibility to these herbs in the tropical climate of Malaysia. When it comes to studies performed globally, *Hibiscus sabdariffa*, *Crataegus aronia*, *Allium sativa*, *Aloe vera* and *Carthamus tinctorius* are some of the more common types of herbs used for hypertension management, which contrasted with the current study [14–16, 18, 19, 29].

Several of the major crude herbs used by patients in the current study have been proven to have antihypertensive properties through *in vitro* and *in vivo* studies. One previous study reveals that a fraction of *C*. *asiatica* which is rich in triterpenoids has antihypertensive effects on hypertensive rats induced by phenylephrine [37]. Besides, bioactive compounds present in *M*. *oleifera* were shown to be potent inhibitors of angiotensin converting enzyme (ACE). Another study indicates that aqueous extracts of *M*. *oleifera* leaves promote blood vessels vasodilation by stimulating the release of endothelium-derived nitric oxide [38, 39]. It is without a doubt, crude herbs can potentially act as antihypertensive agents due to their rich phytochemical content as indicated in past literature. However, previous investigations have also highlighted that when crude herbs are taken concurrently with antihypertensive drugs, they may lead to unwanted herb-drug interactions. The consumption of these herbs may potentiate or antagonise the effects of some antihypertensive drugs, causing changes to the pharmacokinetics of the drug, primarily their metabolic activities [40]. Certain bioactive compounds and aqueous extracts of *M*. *oleifera* were determined to have CYP3A4 and CYP1A2 inhibitory properties respectively [41, 42]. Hence, certain antihypertensive drugs such as amlodipine, is metabolised by CYP3A4 [43]. If the herbs taken concurrently that inhibit the cytochrome P450 enzyme; such reaction may disrupt the metabolism of these drugs, potentially leading to unnecessary herb-drug interactions and adverse effects. Therefore, further investigations on the use of crude herbs for hypertension should be explored through clinical trials.

Overall, the patients in this study had the highest score for social functioning and the lowest score for health change. This is comparable to a previous suburban Malaysian study whereby social functioning showed the highest score while general health had the lowest score among hypertension patients [23]. The reason hypertensive patients showed high scores for social functioning could be due to their ability to adapt and cope with the disease [23]. In this study, there are no significant differences noted for all the nine SF-36 domains between crude herbs users and non-users. Similarly, a previous Malaysian study highlighted that no significant differences were observed in HRQOL between users of CAM and non-users who were cardiovascular disease patients [44]. In a study on breast cancer patients receiving chemotherapy, there were also no significant differences for quality of life when CAM users were compared with non-users [45]. In contrast, a Norwegian study performed on patients with inflammatory bowel disease showed that statistically significant lower scores for SF-36 were seen among CAM users compared with the non-users [46]. Another study highlighted a significant difference in quality of life of coronary heart disease patients between CAM users and non-users, in which CAM users reported improved health status [47].

In the present study, 93.3% of the crude herb users did not inform their physician about the use of herbs. This finding is higher than those of several other studies performed in other countries; for example, Jordan—77.4% [16]; Palestine—68.1% [14]; Sierra Leon—; 85.1% [18]; Nigeria—71.2% [13]; One study reported that the crude herbs users did not inform their physician for two reasons: the doctor did not ask them; patients felt that the doctors would not understand [48]. Essentially, patients and their physicians should practise good communication regarding the use of crude herbs while the former is on a prescribed treatment regime; this will bring about the proper management of hypertension, and any unwanted interactions or effects can be avoided.

In this study, crude herb users cited ease of accessibility and cultural factors as the two main reasons for using crude herbs. When compared with earlier studies, a few of the common reasons for using herbs are as follows: dissatisfaction with the conventional therapy; conventional medicine being too expensive; good experiences in the past when taking herbs; and family traditions [49, 50]. Malaysia is a land of multiple ethnicities; the people of various races have inherited the practice and knowledge pertaining to traditional herbs from the previous generations [51]. The use of herbs in Malaysia is based on observations, rituals, and practical experiences derived from religious beliefs and cultural appropriations [52]. Hence, it is essential for the healthcare providers to delve deeper into the patients’ self-management of using crude herbs in improving their condition.

### Study limitations

This study has several limitations. Firstly, it is designed as a cross-sectional study; hence, a relationship between the outcome and the exposure could not be determined. Besides, the findings may not be representative of the entire country, as the study only includes patients attending a government health clinic in a suburban setting. This survey was only performed in two languages, Bahasa Melayu and English, which limited the inclusion of certain patients who were only well-versed in Cantonese and Tamil. Furthermore, the “others” group is small and small sample sizes limit the accuracy of the inference for the group. Despite these shortcomings, this study highlights the use of crude herbs by primary care patients receiving treatment for hypertension, which may pave a way for future investigations on targeted groups of patients in this field.

## Conclusion

In conclusion, although patients with hypertension seek treatment in health clinics, the integration of crude herbs as a form of self-management was quite common and diverse. The predictors of crude herbs use determined in this study are as follows: having muscle pains, experiencing falls, being Malay or Indian, having secondary education or higher, and having a systolic blood pressure of more than 140 mmHg; this gives us an idea about the possible group of patients who tend to use crude herbs. This vital information can aid the healthcare workers to make informed choices when consulting patients on hypertension management. It is noted that there are no significant differences in HRQOL domains among crude herbs users and non-users. Although patients are adamant about integrating crude herbs as a form of self-management, the effects of doing so have not been properly investigated. This implies that the healthcare staff members need to communicate with the patients regarding the use of crude herbs together with conventional drugs. Education programmes about the proper use of crude herbs, their side effects, contraindication when used with conventional drugs, and overall effectiveness in managing hypertension should be introduced to both the patients and doctors. To achieve this, future studies may include these areas; patient’s needs and perspectives on crude herbs use.

## Supporting information

S1 Fig Logistic regression analysis. (TIF)

S1 Table Health related quality of life domains. (DOCX)

S1 File Survey questionnaire—English version. (PDF)

S2 File Survey questionnaire—Malay version. (PDF)
