# Supplementary figures and images for: Self-management using crude herbs and the health-related quality of life among adult patients with hypertension living in a suburban setting of Malaysia
Source: PLoS One. 2021 Sep 10;16(9):e0257336. doi: 10.1371/journal.pone.0257336 (PMC8432735; doi:10.1371/journal.pone.0257336)

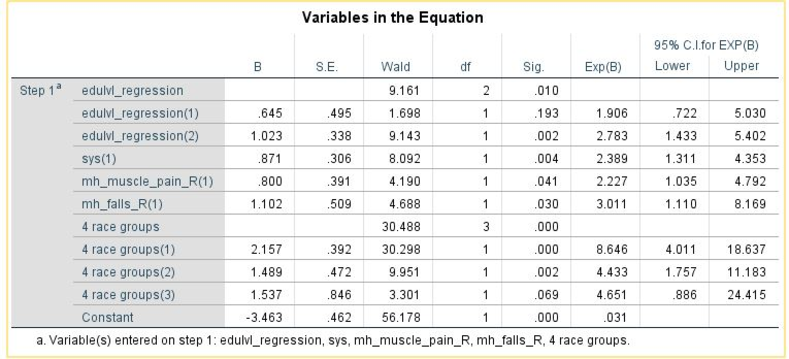

Supplement: S1 Fig — (TIF) [file pone.0257336.s001.tif]
