# Supplementary material for: Self-management using crude herbs and the health-related quality of life among adult patients with hypertension living in a suburban setting of Malaysia
Source: PLoS One. 2021 Sep 10;16(9):e0257336. doi: 10.1371/journal.pone.0257336 (PMC8432735; doi:10.1371/journal.pone.0257336)
Supplement: S1 Table — (DOCX) [file pone.0257336.s002.docx]

**Mann-Whitney U test for health-related quality of life domains**

S1 Table: Health-Related Quality of Life (HRQOL) domains of patients

| **HRQOL domains** | **Patients’ HRQOL scores**  (N = 294) | **Taking crude herbs** | | **Mann-Whitney U value** | **p-value** |
| --- | --- | --- | --- | --- | --- |
|  |  | Yes  (N = 90) | No  (N = 204) |  |  |
|  | Mean ± SD | Mean ± SD | Mean ± SD |  |  |
| Physical Functioning | 78.62 ± 22.50 | 81.17 ± 20.57 | 77.50 ± 23.25 | 8626.5 | -0.831, 0.406 |
| Role limitations due to physical health | 65.56 ± 41.70 | 65.00 ± 43.51 | 65.81 ± 40.98 | 9177.5 | -0.004, 0.997 |
| Role limitations due to emotional problems | 72.52 ± 37.97 | 73.65 ± 35.94 | 72.01 ± 38.90 | 9097.0 | -0.143, 0.887 |
| Energy/fatigue | 60.62 ± 17.17 | 62.94 ± 13.46 | 59.59 ± 18.50 | 8271.5 | -1.359, 0.174 |
| Emotional well-being | 78.07 ± 13.48 | 76.17 ± 15.05 | 78.90 ± 12.67 | 8282.5 | -1.344, 0.179 |
| Social functioning | 91.72 ± 17.14 | 93.24 ± 14.84 | 91.05 ± 18.05 | 8780.5 | -0.785, 0.433 |
| Pain | 84.38 ± 19.89 | 83.74 ± 20.40 | 84.67 ± 19.70 | 8840.0 | -0.531, 0.595 |
| General health | 60.36 ± 13.15 | 60.83 ± 14.64 | 60.15 ± 12.48 | 8490.0 | -1.038,  0.299 |
| Health change | 47.05 ± 17.05 | 47.40 ± 18.60 | 46.89 ± 16.37 | 9107.0 | -0.125, 0.900 |
