# Supplementary material for: Self-management using crude herbs and the health-related quality of life among adult patients with hypertension living in a suburban setting of Malaysia
Source: PLoS One. 2021 Sep 10;16(9):e0257336. doi: 10.1371/journal.pone.0257336 (PMC8432735; doi:10.1371/journal.pone.0257336)
Supplement: S1 File — (PDF) [file pone.0257336.s003.pdf]

Universiti Tunku Abdul Rahman (UTAR)

Wholly owned by UTAR Education Foundation
(Co. No. 578227-M)
DU012(A)

Form Number:
19_______

English Version

QUESTIONNAIRE

UNIVERSITI TUNKU ABDUL RAHMAN
Institute of Postgraduate Studies and Research
Faculty of Science
Master of Science

Title: Crude Herbs Integration into Conventional Care and Its Effect on the Quality Of Life among Patients with Hypertension

---

Dear Participants,

- I am a Master's of Science student from Universiti Tunku Abdul Rahman (UTAR) Kampar Campus. I am doing a study among hypertension patients in government clinics in the Kinta District.
- The purposes of this study are to describe the crude herbs integration by multiethnic primary care patients attending urban and sub-urban health clinics, and to compare quality of life of the hypertension patients who integrate crude herbs with those who do not integrate crude herbs.
- Please read through and consider the information carefully before deciding to participate. Your participation in this study is voluntary and you may withdraw at any point of time. Please read the personal data protection statement and give your consent before participating in this study.
- The information from this study is solely used for research purpose only and all the information obtained will be kept confidential and will not be revealed to any party.
- This questionnaire would take roughly 30 minutes to be completed, and I would greatly appreciate if you could spare some time to complete this questionnaire.
- There are no correct or wrong answers to questions. You are advised to choose option that best describes you or your opinion.
- If you have further queries, please contact Dr Annaletchumy, Telephone number, 05-4688888 ext 4511, email, annal@utar.edu.my.
- Thank you for your participation and your cooperation would be greatly appreciated

---

Universiti Tunku Abdul Rahman (UTAR)

Personal Data Protection Statement

Please be informed that in accordance with Personal Data Protection Act 2010 "PDPA" which come into force on 15 November 2013, Universiti Tunku Abdul Rahman, UTAR is hereby bound to make notice and require consent in relation to collection, recording, storage, usage and retention of personal information.

Notice:

1. The purposes for which your personal data may be used are inclusive but not limited to:-
   - For assessment of any application to UTAR
   - For processing any benefits and services
   - For communication purposes
   - For advertorial and news
   - For general administration and record purposes
   - For enhancing the value of education
   - For educational and related purposes consequential to UTAR
   - For the purpose of our corporate governance
   - For consideration as a guarantor for UTAR staff/ student applying for his/her scholarship/ study loan
2. Your personal data may be transferred and/or disclosed to third party and/or UTAR collaborative partners including but not limited to the respective and appointed outsourcing agents for purpose of fulfilling our obligations to you in respect of the purposes and all such other purposes that are related to the purposes and also in providing integrated services, maintaining and storing records. Your data may be shared when required by laws and when disclosure is necessary to comply with applicable laws.

3. Any personal information retained by UTAR shall be destroyed and/or deleted in accordance with our retention policy applicable for us in the event such information is no longer required.

4. UTAR is committed in ensuring the confidentiality, protection, security and accuracy of your personal information made available to us and it has been our ongoing strict policy to ensure that your personal information is accurate, complete, not misleading and updated. UTAR would also ensure that your personal data shall not be used for political and commercial purposes.

Consent:

1. By submitting this form you hereby authorise and consent to us processing (including disclosing) your personal data and any updates of your information, for the purposes and/or for any other purposes related to the purpose.

2. If you do not consent or subsequently withdraw your consent to the processing and disclosure of your personal data, UTAR will not be able to fulfill our obligations or to contact you or to assist you in respect of the purposes and/or for any other purposes related to the purpose.

3. You may access and update your personal data by writing to us at dhr@utar.edu.my

[ ] I have been notified by you and that I hereby understood, consented and agreed per UTAR above notice.

[ ] I disagree, my personal data will not be processed.

Signature: ______________________________

Name: __________________________________ Date: ______________________________

Section A: Socio Demographic Characteristics

1. Date of birth (day/month/year): __________________

Please tick (√) where appropriate

2. Gender	O Male
	O Female
3. Race	O Malay
	O Chinese
	O Indian
	O Others (please specify): _______________
4. Household income	O No income
	O Below RM3000
	O RM 3000-RM6000
	O Above RM 6000
5. Education level	O No formal education
	O Primary education
	O Secondary education
	O Tertiary education
6. Current employment status	O Employed (Full-time)
	O Employed (Part-time)
	O Housewife/Homemaker
	O Unemployed
	O Retired
	Please state previous employment: _______________

<u>Section B: Disease Details and High Blood Pressure (Hypertension) Management</u>

1. For how long have you been diagnosed with high blood pressure?
   __________ years

2. Has your doctor prescribed you with any medicine to lower blood pressure?
   - O Yes
   - O No
3. Are you taking the blood pressure medication given by your doctor?
   - O Yes
   - O No
4. Are you taking crude herbs to manage your high blood pressure?
   - O Yes
   - O No

<u>Section C: Lifestyle Changes for High Blood Pressure Management</u>

1. Are you practicing the following lifestyle changes to manage your high blood pressure? (You may tick more than one)

- O Trying to reduce body weight
- O Maintaining a healthy body weight
- O Reduce sodium (salt) intake
- O Reduce/stop alcohol consumption
- O Regular physical activity (at least 90 minutes a week)
- O Healthy eating (as advised by your doctor)
- O Reduce/stop smoking
- O Stress management
- O Increased dietary potassium intake (fruits, nuts, vegetables and legumes)
- O Others: _________________________
- O Not practicing any lifestyle changes

Section D: Medical History

1. Besides high blood pressure (hypertension), were you diagnosed with any other diseases?

No.	Medical History	Yes	No	Not sure
1	Asthma	☐	☐	☐
2	Cancer (Type of cancer:______________________)	☐	☐	☐
3	Cardiovascular disease (e.g. *Heart disease*)	☐	☐	☐
4	Diabetes mellitus (*High blood glucose*)	☐	☐	☐
5	Dyslipidemia (*Abnormal amount of lipids (cholesterol/fat) in blood*)	☐	☐	☐
6	Hyperuricaemia *(High level of uric acid in blood)*	☐	☐	☐
7	Kidney disease (*Kidney do not work effectively*）	☐	☐	☐
8	Leukemia	☐	☐	☐
9	Hepatitis, Jaundice, Liver disease	☐	☐	☐
10	Migraine or recurrent headaches	☐	☐	☐
11	Muscle pain（*Due to tension, over work, or muscle injury from exercise*）	☐	☐	☐
12	Obesity（*High body fat. BMI ≥ 30*）	☐	☐	☐
13	Parkinson's disease（*Tremor, muscular rigidity, and slow, imprecise movement*）	☐	☐	☐
14	Peptic ulcer (*Burning stomach pain*)	☐	☐	☐
15	Stroke	☐	☐	☐
16	Thyroid disease	☐	☐	☐
17	Urinary infection (Chronic type: *Infection involving the kidneys, ureters, bladder, or urethra*）	☐	☐	☐
18	Falls/with injury in the past 6 to 12 months (an unexpected event in when a person comes to rest on the ground, floor or lower level).	☐	☐	☐
19	Osteoporosis	☐	☐	☐
20	Others (Please list out):			

Section E: RAND 36-Item Health Survey 1.0 Questionnaire

Choose one option for each questionnaire item. Please tick (√) where appropriate

1. In general, would you say your health is:

○ 1 – Excellent

○ 2 – Very good

○ 3 – Good

○ 4 – Fair

○ 5 – Poor

2. Compared to one year ago, how would you rate your health in general now?

○ 1 – Much better now than one year ago

○ 2 – Somewhat better now than one year ago

○ 3 – About the same

○ 4 – Somewhat worse now than one year ago

○ 5 – Much worse now than one year ago

The following items are about activities you might do during a typical day. Does your health now limit you in these activities? If so, how much?

	Yes, limited a lot	Yes, limited a little	No, not limited at all
3. Vigorous activities, such as running, lifting heavy objects, participating in strenuous sports	○ 1	○ 2	○ 3
4. Moderate activities, such as moving a table, pushing a vacuum cleaner, bowling, or playing golf	○ 1	○ 2	○ 3
5. Lifting or carrying groceries	○ 1	○ 2	○ 3
6. Climbing several flights of stairs	○ 1	○ 2	○ 3
7. Climbing one flight of stairs	○ 1	○ 2	○ 3
8. Bending, kneeling or stooping	○ 1	○ 2	○ 3
9. Walking more than a kilometer	○ 1	○ 2	○ 3
10. Walking several hundred meters	○ 1	○ 2	○ 3
11. Walking one hundred meters	○ 1	○ 2	○ 3
12. Bathing or dressing yourself	○ 1	○ 2	○ 3

During the past 4 weeks, have you had any of the following problems with your work or other regular daily activities as a result of your physical health?

	Yes	No
13. Cut down the amount of time you spent on work or other activities	○ 1	○ 2
14. Accomplished less than you would like	○ 1	○ 2
15. Were limited in the kind of work or other activities	○ 1	○ 2
16. Had difficulty performing the work or other activities (for example, it took extra effort)	○ 1	○ 2

During the past 4 weeks, have you had any of the following problems with your work or other regular daily activities as a result of any emotional problems (such as feeling depressed or anxious)?

	Yes	No
17. Cut down the amount of time you spent on work or other activities	O 1	O 2
18. Accomplished less than you would like	O 1	O 2
19. Didn't do work or other activities as carefully as usual	O 1	O 2

20. During the past 4 weeks, to what extent has your physical health or emotional problems interfered with your normal social activities with family, friends, neighbors, or groups?

O 1 – Not at all
O 2 – Slightly
O 3 – Moderately
O 4 – Quire a bit
O 5 – Extremely

21. How much bodily pain have you had during the past 4 weeks?

O 1 – None
O 2 – Very mild
O 3 – Mild
O 4 – Moderate
O 5 – Severe
O 6 – Very severe

22. During the past 4 weeks, how much did pain interfere with your normal work (including both work outside the home and housework)?

O 1 – Not at all

O 2 – A little bit

O 3 – Moderately

O 4 – Quire a bit

O 5 – Extremely

These questions are about how you feel and how things have been with you during the past 4 weeks. For each question, please give the one answer that comes closest to the way you have been feeling. How much of the time during the past 4 weeks…

	All of the time	Most of the time	A good bit of the time	Some of the time	A little of the time	None of the time
23. Did you feel full of life?	O 1	O 2	O 3	O 4	O 5	O 6
24. Have you been a very nervous person?	O 1	O 2	O 3	O 4	O 5	O 6
25. Have you felt so sad and low in mood that nothing could cheer you up?	O 1	O 2	O 3	O 4	O 5	O 6
26. Have you felt calm and peaceful?	O 1	O 2	O 3	O 4	O 5	O 6
27. Did you have a lot of energy?	O 1	O 2	O 3	O 4	O 5	O 6
28. Have you felt downhearted and depressed?	O 1	O 2	O 3	O 4	O 5	O 6
29. Did you feel worn out?	O 1	O 2	O 3	O 4	O 5	O 6
30. Have you been a happy person?	O 1	O 2	O 3	O 4	O 5	O 6
31. Did you feel tired?	O 1	O 2	O 3	O 4	O 5	O 6

32. During the past 4 weeks, how much of the time has your physical health or emotional problems interfered with you social activities (like visiting with friends, relatives, etc.)?

○ 1 – All of the time

○ 2 – Most of the time

○ 3 – Some of the time

○ 4 – A little of the time

○ 5 – None of the time

How TRUE or FALSE is each of the following statements for you.

	Definitely true	Mostly true	Don't know	Mostly false	Definitely false
33. I seem to get sick a little easier than other people	○ 1	○ 2	○ 3	○ 4	○ 5
34. I am as healthy as anybody I know	○ 1	○ 2	○ 3	○ 4	○ 5
35. I expect my health to get worse	○ 1	○ 2	○ 3	○ 4	○ 5
36. My health is excellent	○ 1	○ 2	○ 3	○ 4	○ 5

Section F: International Complementary and Alternative Medicine (I-CAM-Q) adapted for studies among Malaysian Hypertension Patients

1. Visiting health care providers: Health conditions (high blood pressure) can be attended to by a variety of traditional and complementary health care providers.

Over the **past 12 months**, have you seen any of the following health care providers for high blood pressure treatment and management?			Number of times you saw this provider in **the last 3 months**	How helpful was it for you to see this provider (Please tick only one)			
	Yes	No		Very Helpful	Somewhat Helpful	Not Helpful	Don' t Know
Physician/doctor	☐	☐	_____	☐	☐	☐	☐
Chiropractor	☐	☐	_____	☐	☐	☐	☐
Homeopath provider	☐	☐	_____	☐	☐	☐	☐
Acupuncturist	☐	☐	_____	☐	☐	☐	☐
Herbalist/Naturopath	☐	☐	_____	☐	☐	☐	☐
Spiritual Healer	☐	☐	_____	☐	☐	☐	☐
Chinese Physician	☐	☐	_____	☐	☐	☐	☐
Traditional Malay Medicine Practitioner	☐	☐	_____	☐	☐	☐	☐
Traditional Indian Medicine Practitioner	☐	☐	_____	☐	☐	☐	☐
Others: _______________	☐	☐	_____	☐	☐	☐	☐
Others: _______________	☐	☐	_____	☐	☐	☐	☐

2. Self Help Practices for High Blood Pressure Management.

Have you used any of the following self-help practices to manage high blood pressure in the last 12 months?			Number of times you used this self-help practice in **the last 3 months**	How helpful was it for you to use this self-help practice for high blood pressure management (Please tick only one)			
	Yes	No		Very Helpful	Somewhat Helpful	Not Helpful	Don’ t Know
Siddha home remedy	☐	☐	_____	☐	☐	☐	☐
Yoga	☐	☐	_____	☐	☐	☐	☐
Qigong/ Tai Chi	☐	☐	_____	☐	☐	☐	☐
Meditation	☐	☐	_____	☐	☐	☐	☐
Attended traditional healing ceremony	☐	☐	_____	☐	☐	☐	☐
Praying for own health	☐	☐	_____	☐	☐	☐	☐
Spa therapy	☐	☐	_____	☐	☐	☐	☐
Color vibration therapy	☐	☐	_____	☐	☐	☐	☐
Unani (traditional arab medicine)	☐	☐	_____	☐	☐	☐	☐
Reiki	☐	☐	_____	☐	☐	☐	☐
Phytobiophysics	☐	☐	_____	☐	☐	☐	☐
Pranayama	☐	☐	_____	☐	☐	☐	☐
Crystal therapy	☐	☐	_____	☐	☐	☐	☐
Others : ____________________	☐	☐	_____	☐	☐	☐	☐
Others: ____________________	☐	☐	_____	☐	☐	☐	☐

3. Use of Herbal Medicine and Dietary Supplements, including tablets, capsules and liquids.

For each category below, please list up to three products you have used in the last 12 months.	Do you **currently** use this product?		Number of times you used this product in **the last 3 months**	How helpful did you find this product in managing high blood pressure (Please tick only one)			
	Yes	No		Very Helpful	Somewhat Helpful	Not Helpful	Don' t Know
Crude Herbs							
Please refer to Section G, questions 1 to 4							
Herbal Medicine							
	☐	☐	____	☐	☐	☐	☐
	☐	☐	____	☐	☐	☐	☐
	☐	☐	____	☐	☐	☐	☐
Vitamins, minerals or other supplements							
	☐	☐	____	☐	☐	☐	☐
	☐	☐	____	☐	☐	☐	☐
	☐	☐	____	☐	☐	☐	☐

*For participants who use **crude herbs for high blood pressure management**, please proceed to **Section G** of this questionnaire. For participants who do not use crude herbs, please proceed to **Section H**

Section G: Crude herbs Used by Patients to manage High Blood Pressure

1. Please complete the table below

Crude herbs taken over the **past 12 months**	Herb's part	Preparation methods	Do you currently use this product?	For how long have you been using this product?	Number of times you used this product in **the last 3 months**	How helpful did you find this product in managing high blood pressure (Please tick only one)
			☐			○ Very Helpful<br>○ Somewhat Helpful<br>○ Not Helpful<br>○ Don't Know
			☐			○ Very Helpful<br>○ Somewhat Helpful<br>○ Not Helpful<br>○ Don't Know
			☐			○ Very Helpful<br>○ Somewhat Helpful<br>○ Not Helpful<br>○ Don't Know
			☐			○ Very Helpful<br>○ Somewhat Helpful<br>○ Not Helpful<br>○ Don't Know
			☐			○ Very Helpful<br>○ Somewhat Helpful<br>○ Not Helpful<br>○ Don't Know

2. Is your physician aware of you consuming crude herbs to manage high blood pressure?

- O Yes
- O No (Please give your reason: ____
  ____
  ____)

3. What are your reasons for taking crude herbs to manage high blood pressure? (You may tick **more than one**)

- O Traditional belief on the effectiveness of crude herbs
- O Dissatisfied with conventional medicine
- O Easily accessible
- O Cultural reasons
- O Religious beliefs
- O Worried of the side effects of conventional medicine
- O Others (Please state): ____

4. How do you get the information on the use of crude herbs?
   (You may tick **more than one**)

- O Books/Magazines
- O Newspaper
- O Television
- O Internet websites
- O Pharmacists
- O Doctor
- O Store clerk/sales assistant
- O Specialist in Chinese/Malay/Indian traditional medicine
- O Friend/Neighbours
- O Family or relatives
- O Others (please state:____)

<u>Section H: BMI and Blood Pressure Measurements</u>

1. **Body Mass Index**

   Height : _________________ cm

   Weight : _________________ kg

   BMI : _________________

2. **Arterial blood pressure: _________________**

Thank you for participating in this survey
