# Supplementary material for: Self-management using crude herbs and the health-related quality of life among adult patients with hypertension living in a suburban setting of Malaysia
Source: PLoS One. 2021 Sep 10;16(9):e0257336. doi: 10.1371/journal.pone.0257336 (PMC8432735; doi:10.1371/journal.pone.0257336)
Supplement: S2 File — (PDF) [file pone.0257336.s004.pdf]

Wholly owned by UTAR Education Foundation
(Co. No. 578227-M)
DU012(A)

Form Number:

19_______

Malay Version

BORANG SOAL SELIDIK

UNIVERSITI TUNKU ABDUL RAHMAN
Institute of Postgraduate Studies and Research
Faculty of Science
Master of Science

Tajuk: Integrasi Herba Mentah dalam Penjagaan Konvensional dan Kesannya terhadap Kualiti Hidup dalam kalangan Pesakit Hipertensi

---

Peserta yang dihormati,

- Saya ialah seorang pelajar Sarjana Sains dari Universiti Tunku Abdul Rahman (UTAR) Kampus Kampar. Saya sedang membuat satu kajian dalam kalangan pesakit tekanan darah tinggi di klinik kerajaan tertendu di Daerah Kinta.
- Tujuan kajian ini adalah untuk menggambarkan pengunaan herba oleh pesakit penjagaan utama pelbagai etnik yang datang ke klinik kesihatan bandar dan sub-bandar, dan membandingkan kualiti hidup pesakit tekanan darah tinggi yang mengunakan herba mentah dengan mereka yang tidak menggunakan herba mentah.
- Sila baca dan mempertimbangkan maklumat dengan teliti sebelum membuat keputusan untuk mengambil bahagian dalam kajian ini. Penyertaan anda dalam kajian ini adalah secara sukarela dan anda boleh menarik diri pada bila-bila masa. Sila baca kenyataan perlindungan data peribadi dan memberikan persetujuan anda sebelum mengambil bahagian dalam kajian ini.
- Maklumat daripada kajian ini adalah semata-mata digunakan untuk tujuan penyelidikan sahaja dan semua maklumat yang diperolehi akan dirahsiakan dan tidak akan didedahkan kepada mana-mana pihak
- Soal selidik ini akan mengambil masa kira-kira 30 minit untuk disiapkan, dan saya amat menghargai jika anda boleh meluangkan masa untuk melengkapkan soal selidik ini.
- Tiada jawapan yang betul atau salah untuk setiap soalan. Anda dinasihatkan untuk memilih pilihan paling tepat yang menggambarkan anda atau pendapat anda.
- Jika anda mempunyai sebarang pertanyaan lanjut, sila hubungi Dr Annaletchumy, Nombor telefon, 05-4688888 ext 4511, e-mel, annal@utar.edu.my.
- Terima kasih atas penyertaan anda dan kerjasama anda amat dihargai

---

Universiti Tunku Abdul Rahman (UTAR)

Personal Data Protection Statement

Please be informed that in accordance with Personal Data Protection Act 2010 "PDPA" which come into force on 15 November 2013, Universiti Tunku Abdul Rahman, UTAR is hereby bound to make notice and require consent in relation to collection, recording, storage, usage and retention of personal information.

Notice:

1. The purposes for which your personal data may be used are inclusive but not limited to:-
   - For assessment of any application to UTAR
   - For processing any benefits and services
   - For communication purposes
   - For advertorial and news
   - For general administration and record purposes
   - For enhancing the value of education
   - For educational and related purposes consequential to UTAR
   - For the purpose of our corporate governance
   - For consideration as a guarantor for UTAR staff/ student applying for his/her scholarship/ study loan
2. Your personal data may be transferred and/or disclosed to third party and/or UTAR collaborative partners including but not limited to the respective and appointed outsourcing agents for purpose of fulfilling our obligations to you in respect of the purposes and all such other purposes that are related to the purposes and also in providing integrated services, maintaining and storing records. Your data may be shared when required by laws and when disclosure is necessary to comply with applicable laws.

3. Any personal information retained by UTAR shall be destroyed and/or deleted in accordance with our retention policy applicable for us in the event such information is no longer required.

4. UTAR is committed in ensuring the confidentiality, protection, security and accuracy of your personal information made available to us and it has been our ongoing strict policy to ensure that your personal information is accurate, complete, not misleading and updated. UTAR would also ensure that your personal data shall not be used for political and commercial purposes.

Consent:

1. By submitting this form you hereby authorise and consent to us processing (including disclosing) your personal data and any updates of your information, for the purposes and/or for any other purposes related to the purpose.

2. If you do not consent or subsequently withdraw your consent to the processing and disclosure of your personal data, UTAR will not be able to fulfill our obligations or to contact you or to assist you in respect of the purposes and/or for any other purposes related to the purpose.

3. You may access and update your personal data by writing to us at dhr@utar.edu.my

[ ] I have been notified by you and that I hereby understood, consented and agreed per UTAR above notice.

[ ] I disagree, my personal data will not be processed.

Signature: ______________________________

Name: _________________________________ Date: ______________________________

<u>Bahagian A: Ciri-ciri Socio-Demographi</u>

1. Tarikh Lahir (hari/bulan/tahun): ___________________

Sila tandakan (√) di pilihan yang sesuai

2. Jantina
   - O Lelaki
   - O Perempuan
3. Kaum
   - O Melayu
   - O Cina
   - O India
   - O Asli
   - O Sikh
   - O Lain-lain (sila jelaskan): ________________
4. Gaji
   - O Tiada Pendapatan
   - O Bawah RM3000
   - O RM 3000-RM6000
   - O RM 6000 dan ke atas
5. Tahap Pendidikan
   - O Tidak menerima pendidikan formal dari sekolah
   - O Sekolah rendah
   - O Sekolah menengah
   - O Pendidikan tinggi daripada kolej/universiti
6. Status Pekerjaan Terkini
   - O Bekerja (Penuh-Masa)
   - O Bekerja (Separuh-Masa)
   - O Bekerja Sendiri
   - O Suri rumah tangga
   - O Penganggur
   - O Pesara

     Sila nyatakan pekerjaan anda yang terdahulu:

     ____

Bahagian B: Maklumat berkenaan tekanan darah tinggi (Hipertensi) dan Pengurusannya

7. Untuk berapa lama anda telah disahkan menghidap tekanan darah tinggi?

   ________ tahun

8. Adakah doktor anda ditetapkan anda dengan mana-mana ubat tekanan darah tinggi?
   - O Ya
   - O Tidak
9. Adakah anda mengambil ubat darah tinggi yang ditetapkan oleh doktor anda?
   - O Ya
   - O Tidak
10. Adakah anda mengambil herba mentah untuk menguruskan tekanan darah tinggi anda?
    - O Ya
    - O Tidak

Bahagian C: Perubahan Gaya Kehidupan Untuk Pengurusan Hipertensi

11. Adakah anda mengamalkan perubahan gaya hidup berikut untuk menguruskan tekanan darah tinggi anda? (Anda boleh tanda lebih dari satu)
    - O Cuba untuk mengurangkan berat badan
    - O Mengekalkan berat badan yang sihat
    - O Mengurangkan pengambilan natrium (garam)
    - O Mengurangkan / menghentikan pengambilan alcohol
    - O Bersenam denga kerap (sekurang-kurangnya 90 minit seminggu)
    - O Pemakanan sihat (seperti yang dinasihatkan oleh doktor anda)
    - O Kurangkan / berhenti merokok
    - O Pengurusan stress

- ○ Peningkatan pengambilan kalium pemakanan (buah-buahan, kacang, sayur-sayuran dan kekacang)
- ○ Lain-lain: ______________________________
- ○ Tidak mengamalkan perubahan gaya hidup

Bahagian D: Latar Belakang Perubatan

12. Selain daripada tekanan darah tinggi, adakah anda disahkan menghidap mana-mana penyakit lain?

No	Sejarah Perubatan	Ya	Tidak	Tidak pasti
1	Asma	☐	☐	☐
2	Kanser (Jenis kanser:________________________)	☐	☐	☐
3	Penyakit kardiovaskular (*penyakit jantung*)	☐	☐	☐
4	Kencing Manis/Diabetis	☐	☐	☐
5	Kolesterol / Lemak tinggi	☐	☐	☐
6	Asid Urik tinggi	☐	☐	☐
7	Penyakit buah pinggang	☐	☐	☐
8	Kanser darah/leukemia	☐	☐	☐
9	Hepatitis, Jaundis, Penyakit hati	☐	☐	☐
10	Migrain, Sakit kepala yang berulang-ulang	☐	☐	☐
11	Sakit Otot (*disebabkan oleh ketegangan, kuat kerja, atau kecederaan otot dari senaman*)	☐	☐	☐
12	Obesiti (*Lemak badan tinggi. BMI* $\geq$ *30*)	☐	☐	☐
13	Penyakit Parkinson (*gegaran, ketegaran otot, dan pergerakan yang perlahan*)	☐	☐	☐
14	Ulser peptik (sakit perut)	☐	☐	☐
15	Strok	☐	☐	☐
16	Penyakit tiroid	☐	☐	☐
17	Jangkitan Saluran Kencing (*Jangkitan yang melibatkan buah pinggang, ureter, pundi kencing, atau uretra*)	☐	☐	☐

18	Jatuh/dan cedera dalam tempoh 6 hingga 12 bulan yang lepas (Jatuh tanpa dijangka dan dimana seseorang yang jatuh itu akan mencecah paras lantai atau lebih rendah daripadanya)	☐	☐	☐
19	Osteoporosis	☐	☐	☐
20	Lain-lain (Sila nyatakan):			

Bahagian E: RAND 36-Item Health Survey 1.0 Questionnaire

Pilih satu jawapan untuk setiap soalan berikut. Sila tandakan (√) di mana sesuai.

1. Secara umum, adakah anda akan mengatakan bahawa kesihatan anda adalah:

○ 1 – Paling baik

○ 2 – Sungguh baik

○ 3 – Baik

○ 4 – Sederhana

○ 5 – Tidak baik

2. Berbanding dengan setahun yang lalu, bagaimanakah anda menilai kesihatan anda secara umum sekarang?

○ 1 – Jauh lebih baik sekarang daripada setahun yang lalu

○ 2 – Agak lebih baik sekarang daripada setahun yang lalu

○ 3 – Lebih kurang sama dengan setahun yang lalu

○ 4 – Agak teruk sekarang daripada setahun yang lalu

○ 5 – Lebih teruk sekarang daripada setahun yang lalu

Soalan-soalan berikut adalah mengenai aktiviti yang mungkin akan dilakukan oleh anda pada hari biasa. Adakah anda terhad di dalam sebarang aktiviti berikut kerana keadaan kesihatan anda sekarang? Jika ya, sejauh mana?

	Ya, terbatas dengan banyaknya	Ya, terbatas dengan sedikitnya	Tidak, tidak terbatas sama sekali
3. Aktiviti yang bertenaga dan sihat, seperti berlari, mengangkat barang berat, menyertai sukan yang memerlukan tenaga dan kekuatan	O 1	O 2	O 3
4. Aktiviti sederhana, seperti mengalihkan meja, menyapu, bermain badminton atau bercucuk tanam	O 1	O 2	O 3
5. Mengangkat atau membawa barang-barang runcit	O 1	O 2	O 3
6. Menaiki beberapa larian tangga	O 1	O 2	O 3
7. Menaiki satu larian tangga	O 1	O 2	O 3
8. Membengkok, melutut atau membongkok	O 1	O 2	O 3
9. Berjalan lebih daripada satu kilometer	O 1	O 2	O 3
10. Berjalan beberapa ratus meter	O 1	O 2	O 3
11. Berjalan seratus meter	O 1	O 2	O 3
12. Mandi atau memakai pakaian sendiri	O 1	O 2	O 3

Dalam masa 4 minggu yang lalu, berapa kerapkah anda mengalami sebarang masalah berikut dengan pekerjaan atau aktiviti harian tetap anda yang lain akibat daripada kesihatan fizikal anda?

	Ya	Tidak
13. Mengurangkan jumlah masa yang dihabiskan oleh anda untuk pekerjaan atau aktiviti lain	O 1	O 2

14. Mencapai kurang daripada yang diingini	O 1	O 2
15. Terbatas dari segi jenis pekerjaan atau aktiviti lain	O 1	O 2
16. Mempunyai kesukaran untuk melakukan pekerjaan atau aktiviti lain (misalnya, ia memerlukan usaha yang lebih)	O 1	O 2

Dalam masa 4 minggu yang lalu, berapa kerapkah anda mengalami sebarang masalah berikut dengan pekerjaan atau aktiviti harian tetap anda yang lain akibat daripada sebarang masalah emosi (seperti merasa murung atau bimbang)?

	Ya	Tidak
17. Mengurangkan jumlah masa yang dihabiskan oleh anda untuk pekerjaan atau aktiviti lain	O 1	O 2
18. Mencapai kurang daripada yang diingini	O 1	O 2
19. Melakukan pekerjaan atau aktiviti lain dengan kurang berhati-hati daripada biasa	O 1	O 2

20. Dalam masa 4 minggu yang lalu, sejauh manakah kesihatan fizikal atau masalah emosi mengganggu aktiviti sosial biasa anda bersama keluarga, sahabat handai, jiran-tetangga atau kumpulan?

O 1 – Tidak sama sekali

O 2 – Sedikit

O 3 – Sederhana

O 4 – Agak banyak

O 5 – Amat sangat

21. Dalam masa 4 minggu yang lalu, berapa banyakkah kesakitan yang dialami oleh anda?

O 1 – Tiada

O 2 – Sedikit sangat

○ 3 – Sedikit

○ 4 – Banyak

○ 5 – Agak banyak

○ 6 – Sungguh banyak

22. Dalam masa 4 minggu yang lalu, sejauh manakah kesakitan telah mengganggu pekerjaan

○ 1 – Tidak sama sekali

○ 2 – Sedikit

○ 3 – Sederhana

○ 4 – Agak banyak

○ 5 – Amat sangat

Soalan-soalan ini adalah mengenai perasaan dan keadaan anda dalam masa 4 minggu yang lalu. Untuk setiap soalan, sila berikan satu jawapan yang paling hampir dengan keadaan perasaan anda. Dalam masa 4 minggu yang lalu, berapa kerapkah…

	Setiap masa	Kebanya kan masa	Banyak masa	Kadang kala	Sedikit masa	Tiada sama sekali
23. Adakah anda merasa penuh bersemangat	○ 1	○ 2	○ 3	○ 4	○ 5	○ 6
24. Pernahkah anda merasa sungguh gementar??	○ 1	○ 2	○ 3	○ 4	○ 5	○ 6
25. Pernahkah anda merasa sungguh sedih hingga tiada apa pun yang dapat menceriakan anda?	○ 1	○ 2	○ 3	○ 4	○ 5	○ 6
26. Pernahkah anda merasa tenang dan aman?	○ 1	○ 2	○ 3	○ 4	○ 5	○ 6
27. Adakah anda sungguh bertenaga?	○ 1	○ 2	○ 3	○ 4	○ 5	○ 6

28. Pernahkah anda merasa sedih dan murung?	O 1	O 2	O 3	O 4	O 5	O 6
29. Adakah anda merasa sangat letih?	O 1	O 2	O 3	O 4	O 5	O 6
30. Pernahkah anda merasa gembira?	O 1	O 2	O 3	O 4	O 5	O 6
31. Adakah anda merasa letih?	O 1	O 2	O 3	O 4	O 5	O 6

32. Dalam masa 4 minggu yang lalu, berapa kerapkah kesihatan fizikal atau masalah emosi telah mengganggu aktiviti sosial anda (seperti melawat sahabat-handai, sanak-saudara, dll.)?

O 1 – Setiap masa

O 2 – Kebanyakan masa

O 3 – Kadang-kala

O 4 – Sedikit masa

O 5 – Tiada sama sekali

Berapa BENAR atau SALAH setiap penyataan berikut untuk anda?

	Sungguh benar	Kebanyakan-nya benar	Tidak tahu	Kebanyakan-nya tidak benar	Sungguh tidak benar
33. Saya kelihatan lebih mudah jatuh sakit daripada orang lain	O 1	O 2	O 3	O 4	O 5
34. Saya sihat seperti orang lain yang saya kenali	O 1	O 2	O 3	O 4	O 5
35. Saya menjangkakan kesihatan saya akan menjadi lebih teruk	O 1	O 2	O 3	O 4	O 5
36. Kesihatan saya adalah sungguh baik	O 1	O 2	O 3	O 4	O 5

Bahagian F: "International Complementary and Alternative Medicine" (I-CAM-Q) disesuaikan untuk kajian dalam kalangan Pesakit Hipertensi Malaysia

1. Melawat penyedia penjagaan kesihatan: Keadaan kesihatan (tekanan darah tinggi) boleh dirawati oleh pelbagai pegawai kesihatan tradisional dan komplementari.

Dalam tempoh 12 bulan yang lepas, adakah anda melihat mana-mana penyedia penjagaan kesihatan yang berikut untuk rawatan dan pengurusan tekanan darah tinggi.			Bilangan kekerapan anda melawat penyedia ini dalam 3 bulan lepas	Adakah perjumpaan dengan penyedia tersebut berfaedah kepada anda? (Sila tandakan satu jawapan sahaja)			
	Ya	Tidak		Sangat Berfaedah	Agak Berfaedah	Tidak Berfaedah	Tidak Pasti
Doktor	☐	☐	______	☐	☐	☐	☐
Chiropractor	☐	☐	______	☐	☐	☐	☐
Penyedia Homeopath	☐	☐	______	☐	☐	☐	☐
Penyedia Akupunktur	☐	☐	______	☐	☐	☐	☐
Saudagar ubat herba	☐	☐	______	☐	☐	☐	☐
Penyembuh rohani	☐	☐	______	☐	☐	☐	☐
Penyedia perubatan Cina	☐	☐	______	☐	☐	☐	☐
Penyedia perubatan Melayu	☐	☐	______	☐	☐	☐	☐
Penyedia perubatan India	☐	☐	______	☐	☐	☐	☐

Lain-lain: ________________	☐	☐	_____	☐	☐	☐	☐
Lain-lain: ________________	☐	☐	_____	☐	☐	☐	☐

2. Amalan Mengurus Tekanan Darah Tinggi dengan sendiri.

Adakah anda pernah menggunakan mana-mana daripada amalan berikut untuk menguruskan tekanan darah tinggi dalam tempoh **12 bulan** yang lepas?			Bilangan kekerapan anda mengamalkan amalan ini dalam tempoh **3 bulan** yang lepas	Adakah amalan tersebut berfaedah kepada anda dalam mengurus tekanan darah tinggi? .(Sila tandakan satu jawapan sahaja)			
	Ya	Tidak		Sangat Berfaedah	Agak Berfaedah	Tidak Berfaedah	Tidak Pasti
Siddha	☐	☐	_____	☐	☐	☐	☐
Yoga	☐	☐	_____	☐	☐	☐	☐
Qigong/ Tai Chi	☐	☐	_____	☐	☐	☐	☐
Meditasi	☐	☐	_____	☐	☐	☐	☐
Menghadiri upacara penyembuhan tradisional	☐	☐	_____	☐	☐	☐	☐
Berdoa untuk kesihatan sendiri	☐	☐	_____	☐	☐	☐	☐
Terapi Spa	☐	☐	_____	☐	☐	☐	☐
Terapi Warna	☐	☐	_____	☐	☐	☐	☐
Unani	☐	☐	_____	☐	☐	☐	☐
Reiki	☐	☐	_____	☐	☐	☐	☐

Phytobiophysics	☐	☐	_____	☐	☐	☐	☐
Pranayama	☐	☐	_____	☐	☐	☐	☐
Terapi kristal	☐	☐	_____	☐	☐	☐	☐
Lain-lain : ________________	☐	☐	_____	☐	☐	☐	☐
Lain-lain: ________________	☐	☐	_____	☐	☐	☐	☐

3. Penggunaan Perubatan Herba dan Suplemen tambahan, termasuk tablet, kapsul dan cecair.

Untuk setiap kategori berikut, sila nyatakan 3 jenis produk yang anda telah gunakan dalam tempoh **12 bulan** yang lepas.	Andakah anda menggunakan produk in sekarang?		Bilangan kekerapan anda menggunakan produk in dalam tempoh 3 bulan yang lepas	Adakah produk ini berfaedah kepada anda dalam mengurus tekanan darah tinggi? (sila tandakan satu jawapan sahaja)			
	Ya	Tidak		Sangat Berfaedah	Agak Berfaedah	Tidak Berfaedah	Tidak Pasti
Herba Mentah							
Sila rujuk bahagian G, soalan 1 higgna 4							
Perubatan Herba							
	☐	☐	_____	☐	☐	☐	☐
	☐	☐	_____	☐	☐	☐	☐
	☐	☐	_____	☐	☐	☐	☐
Vitamin, mineral atau Suplemen							
	☐	☐	_____	☐	☐	☐	☐
	☐	☐	_____	☐	☐	☐	☐

	☐	☐	_____	☐	☐	☐	☐

* Bagi peserta yang menggunakan herba mentah untuk rawatan tekanan darah tinggi, sila terusan ke Bahagian G soal selidik ini. Bagi peserta yang tidak menggunakan herba mentah sila teruskan ke Bahagian H

Bahagian G: Penggunaan Herba Mentah Untuk Pengurusan Tekanan Darah Tinggi

1. Sila lengkapkan jadual di bawah

Herba Mentah yang diambil dalam tempoh 12 bulan yang lepas	Bahagian herba yang digunakan	Cara penyediaan	Adakah anda menggunakan herba ini sekarang?	Untuk berapa lama anda telah mula menggunkan herba ini?	Bilangan kekerapan anda menggunakan herba ini dalam tempoh 3 bulan yang lepas	Adakah herba ini berfaedah kepada anda dalam mengurus tekanan darah tinggi? (sila tandakan satu jawapan sahaja)
			☐			○ Sangat berfaedah<br>○ Agak berfaedah<br>○ Tidak berfaedah<br>○ Tidak pasti
			☐			○ Sangat berfaedah<br>○ Agak berfaedah<br>○ Tidak berfaedah<br>○ Tidak pasti
			☐			○ Sangat berfaedah<br>○ Agak berfaedah<br>○ Tidak berfaedah<br>○ Tidak pasti
			☐			○ Sangat berfaedah<br>○ Agak berfaedah<br>○ Tidak berfaedah<br>○ Tidak pasti
			☐			○ Sangat berfaedah<br>○ Agak berfaedah<br>○ Tidak berfaedah<br>○ Tidak pasti

2. Adakah doktor anda sedar bahawa anda sedang mengambil herba mentah untuk mengurus tekanan darah tinggi?

- ○ Ya
- ○ Tidak (Sila berikan alasan anda: ____

3. Apakah sebab anda mengambil herba mentah untuk mengurus tekanan darah tinggi? (Anda boleh memilih **lebih daripada satu** jawapan)

- ○ Kepercayaan tradisional mengenai keberkesanan herba mentah
- ○ Tidak puas hati dengan perubatan konvensional (diberi oleh klinik/hospital)
- ○ Mudah diperolehi
- ○ Sebab budaya
- ○ Kepercayaan agama
- ○ Bimbang terhadap kesan-kesan sampingan perubatan konvensional
- ○ Lain-lain (Sila nyatakan): ____________________________

4. Dari manakah anda mendapat maklumat mengenai pengunaan herba mentah untuk pengurusan tekanan darah tinggi? (Anda boleh memilih **lebih daripada satu** jawapan)

- ○ Buku/Majalah
- ○ Surat Khabar
- ○ Televisyen
- ○ Laman Web Internet
- ○ Ahli Farmasi
- ○ Doktor
- ○ Kerani stor/ jurujual
- ○ Pakar perubatan tradisional Cina/Melayu/India
- ○ Kawan atau jiran
- ○ Keluarga atau saudara-mara
- ○ Lain-lain (sila nyatakan:____)

Bahagian H: Indeks Jisim Tubuh (BMI) dan Ukuran Tekanan Darah

1. **Indeks Jisim Tubuh**

   Tinggi : ________________cm

   Berat : ________________kg

   BMI : ________________

2. **Tekanan Darah Arteri : ________________________**
